# The Association of Adiposity Indices and Plasma Vitamin D in Young Females in Saudi Arabia

**DOI:** 10.1155/2016/1215362

**Published:** 2016-07-25

**Authors:** Lubna Ibrahim Al Asoom

**Affiliations:** Physiology Department, College of Medicine, University of Dammam (UOD), P.O. Box 1982, Dammam 31441, Saudi Arabia

## Abstract

*Background*. Vitamin D deficiency is a global health problem. Some evidences indicate its association with metabolic syndrome, type II diabetes, and cardiovascular diseases. In the current study we aim to study the association of vitamin D level and indicators of adiposity in young Saudi females.* Subjects and Methods*. 87 young healthy Saudi females were recruited from University of Dammam, Dammam, Saudi Arabia. Each subject filled vitamin D questionnaire and had exercise stress test to determine VO_2_ peak. Body weight, BMI, waist and hip circumference, and ratios were determined. Blood was analyzed for 25-OH vitamin D, glucose, triglycerides, total cholesterol, and differential cholesterol.* Results*. 25-OH vitamin D/body weight was negatively associated with waist circumference and waist/stature ratio. No significant difference was found between the groups of BMI with regard to the data of questionnaire or 25-OH vitamin D/body weight. Obese and overweight subjects had lower VO_2_ peak.* Conclusion*. In young Saudi females we found that the relative value of vitamin D to body weight is a better indicator of vitamin D status particularly in obese subjects and it is negatively associated with adiposity measures of waist circumference and waist/stature ratio.

## 1. Introduction

Vitamin D deficiency is currently one of the major health problems worldwide [1, 2]. The prevalence is growing fast affecting not only the sun deficient countries but also the sunniest lands in the globe [3]. Saudi Arabia is one of the countries that showed reports of high incidence of vitamin D deficiency that affects wide range of different age groups and both sexes. In one cross-sectional study in Riyadh done on healthy adults, 29% of the subjects were vitamin D deficient and about 23% insufficient [4]. Similarly, in another study in Qassim, 28% were vitamin D deficient and 39% were insufficient [5]. Hussain et al. reported high incidence of vitamin D deficiency in Saudi population and emphasized the female gender as a risk factor [6].

Vitamin D is a derivative of a precursor known as dehydrocholesterol present in the subcutaneous tissue. It is initially activated by the exposure to sun light and converted to cholecalciferol. Consequent activation takes place in the liver and kidneys by two stages of hydroxylation. The active form of vitamin D is 1,25-OH vitamin D which is rapidly transferred from circulation to the tissue to exert its multiple actions through the widespread distributed vitamin D receptors VDR [7]. Vitamin D can also be provided through diet particularly the known rich sources such as dairy products, eggs, and fish [8].

Vitamin D level is assessed by measuring the metabolite 25-OH vitamin D. The latter metabolite owned longer bioavailability than 1,25-OH vitamin D [9]. Several clinically approved methods are used to measure 25-OH vitamin D such as chemiluminescence immunoassay (CLIA), enzyme-linked immunosorbent assay (ELISA), and radioimmunoassay (RIA). However high performance liquid chromatography (HPLC) might be the most reliable and yielded the highest values [10].

The significance of vitamin D deficiency was highlighted due to its association with multiple chronic diseases such as metabolic syndrome, diabetes type II, cardiovascular diseases, and cancer [9, 11].

As a trial to explain the nature and mechanism of the relationship between vitamin D deficiency and metabolic syndrome, some studies aimed to study the correlation of 25-OH vitamin D level and markers of adiposity such as BMI, waist circumference, and % of body fat. However, different reports yielded conflicting results [12, 13]. Some authors related the controversy to racial, gender, or age differences [14, 15].

Therefore, in the current study we aim to estimate the level of 25-OH vitamin D in young Saudi females using the best described method, that is, HPLC, and to explore the nature of the relationship between 25-OH vitamin D and markers of adiposity including body weight, BMI, waist circumference, hip circumference, waist/hip, and waist/stature ratios specifically in the females of this ethnic group.

## 2. Subjects and Methods

The current cross-sectional study was approved by the Institutional Review Board at University of Dammam (IRB certificate number 2015-01-065), Dammam, Saudi Arabia, following the principles of Helsinki Declaration [16].

87 young Saudi females were recruited from the female only campus of University of Dammam in the period from October to April 2015 excluding exam periods.

The recruited subjects were healthy and young (mean age: 20.8 ± 2.4 years). They had no history of endocrine diseases, diabetes, hypertension, and liver or renal diseases. They were not receiving any hormone replacement, multivitamin, or vitamin D supplements for at least the last six weeks. None of the participants was pregnant or lactating during the last two years.

The inclusion and exclusion criteria adopted in this study aimed to limit the effect of the confounding factors. There were no specific criteria for BMI and level of physical activity.

Each subject was interviewed and full medical history was obtained including past medical, surgical, hospital admission, drug history, and family history. Then vitamin D questionnaire was filled. All study procedures were explained (blood extraction and exercise stress test) and written informed consent was obtained.

### 2.1. Vitamin D Questionnaire

Each participant filled a questionnaire designed to estimate the intake of vitamin D through diet, sun exposure, and physical activity. Frequency intake of the following articles was estimated: milk, yoghurt, cheese, oil and butter, eggs, meat and fish, carbonated drinks, and vitamin D fortified drinks. Sun exposure was assessed according the following dimensions: duration, frequency, and body extent of exposure. Physical activity was estimated by home activity, time spent using computer and other electronics, walking time, frequency of stairs climbing, and engagement in exercise activity (duration and frequency).

The questionnaire is a modified version of vitamin D questionnaire used by Bolek-Berquist et al. [17]. The questionnaire is provided as supplementary file (see Supplementary Material available online at http://dx.doi.org/10.1155/2016/1215362). Scores were not visible for the subjects.

### 2.2. Anthropometric Data

Body weight was determined for each subject while wearing light clothing using a portable platform scale with accuracy within 0.1 Kg. Height was assessed without shoes using a stadiometer scale with accuracy to the nearest 0.25 cm. BMI was calculated using the formula weight Kg/height M^2^. Central obesity was assessed by waist circumference (at the level of umbilicus using unstretchable measuring tape while wearing light clothes), hip circumference (determined as the largest diameter of the hip), and the ratios waist/hip and waist/stature (height).

### 2.3. Exercise Stress Test

Exercise stress test was used to estimate VO_2_ peak which is the maximum oxygen utilization/min that reflects the cardiorespiratory fitness.

Each subject performs exercise stress test on treadmill following Bruce protocol [18]. In Bruce protocol, the subject starts with speed 1.7 mph and 10% grade. Consequently, both speed and inclination are increased every three minutes until exhaustion.

All participants were encouraged to continue exercise till exhaustion.

VO_2_ peak was calculated by Pollock formula for active and sedentary women using the total run time (*T*):

(1) $$\begin{array}{c} Pollock\;\;{VO}_{2}\;\;peak=4.38\times T-3.9; \end{array}$$

see [19].

### 2.4. Vitamin D Determination

Blood samples were collected by a nurse through venipuncture in the day time period 12-1 pm for all subjects. Blood was collected in EDTA vacutainers and centrifuged and the plasma kept at −80°C for a maximum period of one week before analysis.

Plasma 25-OH vitamin D was measured using ClinRep HPLC Complete Kit (Recipe, Germany).

The procedure began with the extraction of vitamin D from plasma using the precipitant reagent provided by the manufacturer. The analysis was conducted using Waters 2535 quaternary gradient pump system, 2489 UV/Vis detector, and 2707 autosampler according to the manufacturer instruction. In each run, ClinTest standard, calibrator, and quality controls were used to verify the instrument method. Linearity is 2.6–500 *μ*g/L, detection limit is 1.0 *μ*g/L, and quantitation limit is 2.6 *μ*g/L. Inter- and intra-assay coefficient of variation are 3.1–4.7 and 2.3–4.9, respectively.

### 2.5. Statistical Analysis

SPSS software version 20 was used. All data were expressed as mean ± SD and analyzed as normally distributed data according to the result of Kolmogorov-Smirnov *Z* test.

The study sample was divided into 4 groups based on BMI value according to the WHO definition and classification of obesity.

ANOVA and LSD* post hoc* test were used to compare 25-OH vitamin D, 25-OH vitamin D/body weight, data of vitamin D questionnaire, VO_2_ peak, and biochemical tests among different categories of BMI.

Pearson bivariate correlation was used to find out possible correlation between plasma 25-OH vitamin D level and adjusted vitamin D/body weight from one side and indices of adiposity including body weight, BMI, waist and hip circumference and waist/hip, waist/stature ratios, data of vitamin D questionnaire, biochemical test including random blood glucose level, total cholesterol, HDL, LDL, total cholesterol/HDL, and LDL/HDL ratios from the other side. Level of significance was set at *α* < 0.05.

## 3. Results

General characteristics of the participants are listed in Table 1, including age, anthropometric data, and 25-OH vitamin D level.

According to WHO classification, the sample was stratified based on BMI value and yielded the following categories: 12.5% were underweight, 53% were normal, 19.3% were overweight, and 8% were obese (Table 2). When the waist circumference was used as the basis of classification, 77.3% were normal, 17% were overweight, and 5.7% were obese (Table 3).

**Figure 1:**
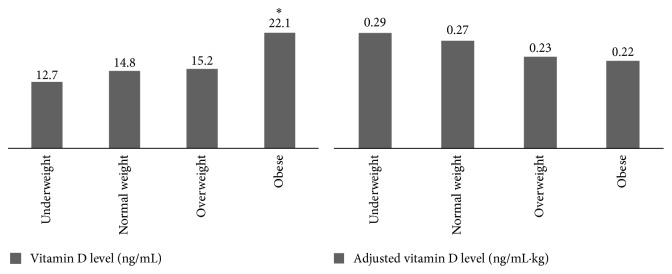
Comparison of vitamin D level and adjusted vitamin D/body weight among different groups of BMI. ^*∗*^ *P* < 0.05.

Plasma 25-OH vitamin D level was significantly higher in obese group than the other groups using ANOVA and LSD* post hoc* test. However when 25-OH vitamin D was corrected to body weight no significant difference was found between the groups (Figure 1).

**Figure 2:**
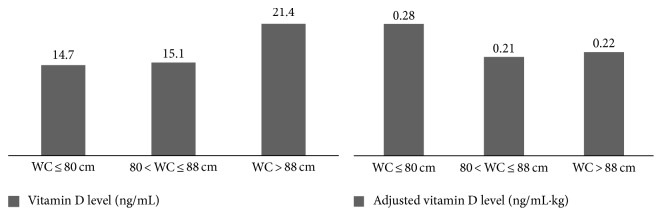
Comparison of plasma vitamin D level and adjusted vitamin D/body weight among different groups of waist circumference WC.

When 25-OH vitamin D and adjusted vitamin D/body weight were compared between the groups of waist circumference no significant difference was found between any of the groups (Figure 2).

There was also no significant difference between BMI groups with regard to the data obtained from the questionnaire. Those data include the frequency and amount of factors that increase vitamin D intake such as diet, sun exposure, and physical activity (Table 4).

### 3.1. VO_2_ Peak

**Figure 3:**
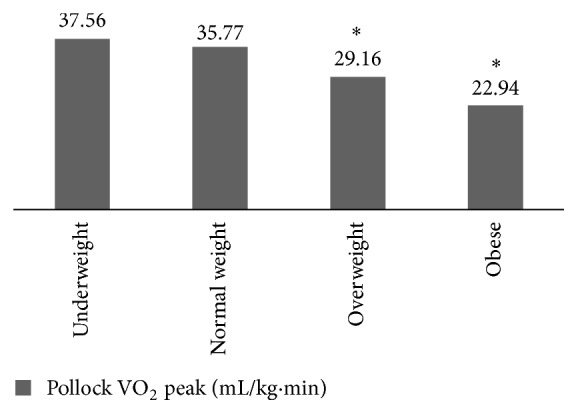
Comparison of cardiorespiratory fitness (VO_2_ peak) among the groups of BMI. ^*∗*^ *P* < 0.05.

Cardiorespiratory fitness represented by VO_2_ peak was significantly lower in overweight and obese compared to normal weight and underweight subjects (Figure 3).

### 3.2. Biochemical Tests

The comparison of biochemical tests including random blood glucose, triglycerides, total cholesterol, HDL, LDL, total cholesterol/HDL, and LDL/HDL among BMI groups yielded significantly higher triglycerides and total cholesterol/HDL in obese compared to normal weight subjects. HDL was significantly higher in underweight groups compared to the other groups (Table 5).

### 3.3. Bivariate Pearson Correlation

**Figure 4:**
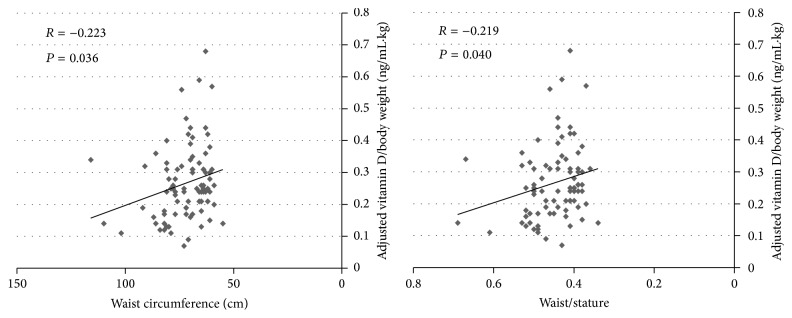
Correlation of adjusted vitamin D level/body weight and waist circumference and waist/stature ratio.

Bivariate Pearson correlation revealed a significant positive correlation between 25-OH vitamin D and BMI and waist circumference. The positive correlation of 25-OH vitamin D and BMI and waist circumference persisted with exclusion of the underweight group (data are not shown). When 25-OH vitamin D is adjusted to body weight, bivariate Pearson correlation showed negative correlation between the relative 25-OH vitamin D/body weight and waist circumference and waist/stature ratio (Figure 4). Similarly, the latter relationship persisted after exclusion of underweight group. No significant correlation was demonstrated between the relative vitamin D/body weight and other measures of adiposity or plasma level of glucose, triglycerides, total cholesterol, or deferential cholesterol parameters.

## 4. Discussion

The current study aimed to find out the possible relationship between plasma 25-OH vitamin D and the markers of body adiposity including BMI, waist and hip circumference, and waist/hip and waist/stature ratios in young Saudi females. The relationship between vitamin D and markers of adiposity was studied recently and attracted the attention of multidisciplinary researchers. It gains its significance from the notion that links vitamin D deficiency with the pathophysiology of common widespread and health threatening diseases such as metabolic syndrome, type II diabetes, cardiovascular disorders, and cancer [20, 21]. However, conflicting data from different populations were reported [22].

In our present study, plasma 25-OH vitamin D showed a significant positive correlation with BMI and waist circumference. This relationship was previously reported in the same geographical location of eastern province of Saudi Arabia by Al-Elq et al. [23]. Al-Elq et al. demonstrated that vitamin D level was positively related to BMI in young females with mean BMI = 23.96. However, when our reported correlation is compared to other studies from different global regions, conflicting data were found. In a study performed by Gilsanz et al. in 90 young healthy females aged 16–22, negative correlation between 25-OH vitamin D and BMI was demonstrated. The BMI of Gilsanz et al.'s subjects was relatively higher than our present study [24]. Similarly, in a relatively large study performed in a metabolic clinic in Norway, negative correlation was found between BMI and 25-OH vitamin D level. Their results might be attributed to the structure of the studied population which was composed of male and female patients with some element of metabolic syndrome and the higher BMI of their female sample than our sample (mean BMI = 31.8 ± 6.28 compared to ours 23.0 ± 4.8), with 88% overweight and obese compared to 27% in our sample. Interestingly, their female subjects with morbid obesity ≥40 had higher 25-OH vitamin D than men with the same BMI category [25]. Therefore, we might propose that the correlation nature between 25-OH vitamin D and BMI might be influenced by structure of the recruited sample with regard to gender and BMI range.

Moreover, in a cross-sectional study of Cypriot adolescent, a U-shaped relationship was suggested between BMI and vitamin D which was reflected by significantly low vitamin D in underweight and obese subjects [3], while in the large HELENA study in Europe in male and female adolescents, no significant correlation was found between vitamin D level and BMI [26].

In order to find out an explanation of our current positive correlation of vitamin D and obesity (BMI and waist circumference), the participants were compared with regard to their exposure to vitamin D sources such as vitamin D rich diet, sun exposure, physical activity, and fitness. We have speculated initially that our overweight and obese young and educated ladies might be eager for healthy diet and exercise more than normal weight candidates which ultimately culminate in enhancement of their vitamin D level, reflected by the positive correlation with BMI. However, that hypothesis was abolished by the insignificant difference in diet, sun exposure, and physical activity scores among the groups of BMI. In addition, overweight and obese were significantly less fit than the normal weight subjects according to their maximum fitness level measured by exercise test and the estimated VO_2_ peak.

Compared to Al-Elq et al. study [23], we have included in our study additional measures of adiposity including waist and hip circumference and waist/hip and waist/stature ratios and aimed to limit the confounding factors of age and gender. However, the positive correlation of 25-OH vitamin D and adiposity measures persisted. Unfortunately, we were unable to estimate plasma PTH due to technical flaws.

Thereafter, we aimed to focus on the relative level of plasma vitamin D to body weight of the current recruited subjects. Consequently, a significant negative correlation was found between the relative vitamin D level to body weight and waist circumference and waist circumference/stature ratio. Therefore, we suggest that the relative vitamin D/body weight is a better indicator of the status of vitamin D level. In addition, the value of relative vitamin D level to body weight was slightly lower in obese and overweight subjects but insignificant. This lack of significance might be attributed to the small sample size of obese subgroup.

Negative correlation of the relative vitamin D/body weight with waist circumference and waist/stature ratio specifically might highlight the significance of the association of vitamin D level with measures of adiposity providing that waist circumference and waist/stature ratio are better indicators of adiposity than BMI [14, 27].

Last but not least, although the correlation coefficients (r) of the relationship of the relative vitamin D/body weight and waist circumference and waist stature ratio support the negative association of vitamin D and adiposity, they are of small value. This might indicate the existence of other confounding factors. First, this might be explained by the diversity of VDR polymorphism in different racial groups [28]. Second, this might reflect the interaction with other hormones involved in the regulation of adipose tissue synthesis and turnover such as PTH, leptin, and adiponectin [29, 30].

## 5. Conclusion

We conclude that the relative vitamin D/body weight might be a better indicator of the vitamin D status particularly in obese subjects. This statement might be supported by the demonstrated negative correlation of relative vitamin D/body weight with adiposity measures, that is, waist circumference and waist/stature ratio in the absence of similar correlation with plasma vitamin D. Therefore, we recommend the implementation of the current measure of relative vitamin D/body weight in future studies and assessment to minimize the conflict and diversity of the results.

## Supplementary Material

This is Vitamin D questionnaire submitted to all subjects to estimate the intake of vitamin D rich food articles, sun exposure, and physical activity. The questionnaire is designed to provide cummulative scores for each segment, however the scores were not visible to the subject.

## Figures and Tables

**Table 1:** Age, anthropometric data, and vitamin D level of 87 young Saudi females.

Character	Mean ± SD	Normal international values for females
Age	20.8 ± 2.4 Years	
Weight	58.1 ± 14.8 Kg	
Height	158.3 ± 6.3 cm	
BMI	23.0 ± 4.8 Kg/m^2^	20–25 Kg/m^2^
Waist circumference	72.2 ± 10.8 cm	<80 cm
Hip circumference	97.5 ± 11.5 cm	
Waist/hip	0.73 ± 0.05	<0.80
Waist/stature	0.44 ± 0.06	<0.5
Plasma 25-OH vitamin D (ng/mL)	15.19 ± 7.19	≥30 ng/mL

**Table 2:** BMI sample subgroups following WHO classification.

	Number of subjects	Percentage %
BMI < 18.5 underweight	11	12.5
18.5 ≤ BMI < 25 normal weight	53	60.2
25 ≤ BMI < 30 overweight	17	19.3
30 ≤ BMI obese	7	8.0

**Table 3:** Waist circumference sample subgroups following WHO classification.

	Number of subjects	Percentage
WC ≤ 80 cm	68	77.3%
88 ≥ WC > 80 cm	15	17%
WC > 88 cm	5	5.7%

**Table 4:** Comparison of diet, sun exposure, and physical activity as evaluated by vitamin D questionnaire among the groups of BMI.

Questions	Underweight *N* = 11	Normal weight *N* = 53	Overweight *N* = 17	Obese *N* = 7
*Diet category*				
Milk amount	1.9 ± 1.6	2.5 ± 1.37	2.6 ± 1.2	2.6 ± 1.4
Milk with coffee	2.4 ± 1.4	2.6 ± 1.4	1.8 ± 1.6	2.4 ± 1.7
Cereals with milk	1.6 ± 1.3	1.7 ± 1.2	2.0 ± 0.9	1.7 ± 1.0
Milk score	7.2 ± 2.7	8.2 ± 2.9	7.7 ± 3.3	8.1 ± 2.7
Yoghurt	2.1 ± 1.3	2.2 ± 1.3	2.1 ± 1.3	2.6 ± 0.8
Oil and butter	2.7 ± 1.7	2.7 ± 1.3	3.3 ± 0.7	2.4 ± 1.7
Cheese	3.4 ± 0.7	3.0 ± 0.9	2.7 ± 0.9	3.0 ± 0.6
Bread	3.6 ± 0.7	3.5 ± 0.6	3.5 ± 0.6	3.7 ± 0.5
Meat and fish	3.2 ± 0.6	3.1 ± 0.5	2.9 ± 0.6	2.9 ± 1.3
Eggs	2.5 ± 0.8	2.4 ± 1.0	2.1 ± 0.9	3.0 ± 0.0
Diet score	24.7 ± 4.8	25.1 ± 4.2	24.3 ± 4.9	25.7 ± 3.9
Carbonated drinks	1.0 ± 0.8	0.9 ± 1.1	1.8 ± 0.9	1.6 ± 1.4
Vitamin D fortified drinks	1.3 ± 1.4	1.3 ± 1.4	1.1 ± 1.1	0.1 ± 0.4
Sun exposure	3.1 ± 1.6	3.0 ± 2.2	2.2 ± 1.7	2.9 ± 2.7
Screen time (T.V and computer)	8.6 ± 4.7	8.4 ± 4.7	10.0 ± 4.4	9.3 ± 2.9
Stair climbing	3.7 ± 2.8	3.6 ± 2.7	2.9 ± 1.9	2.3 ± 1.1.5
Home activity	8.1 ± 3.0	8.5 ± 3.8	7.4 ± 3.4	8.6 ± 4.0
Leisure exercise activity	5.7 ± 1.2	5.6 ± 2.0	4.8 ± 1.9	5.0 ± 2.1

**Table 5:** Comparison of biochemical test results among the groups of BMI.

Biochemical test	Underweight *N* = 11	Normal weight *N* = 53	Overweight *N* = 17	Obese *N* = 7
Random glucose (mg/dl)	92.73 ± 12.73	87.90 ± 13.10	92.53 ± 12.51	94.40 ± 9.23
Triglycerides (mg/dl)	59.45 ± 12.41	77.90 ± 33.56	77.67 ± 29.43	158.20 ± 128.24^*∗∗*^
Total cholesterol (mg/dl)	171.90 ± 21.25	166.35 ± 29.08	162.73 ± 28.56	184.20 ± 53.64
HDL (mg/dl)	56.36 ± 12.02^*∗∗*^	48.44 ± 7.44	46.07 ± 6.50	41.80 ± 4.21
LDL (mg/dl)	59.36 ± 12.05	64.92 ± 16.25	64.20 ± 18.61	69.80 ± 28.10
Total cholesterol/HDL	3.14 ± 0.59	3.47 ± 0.60	3.56 ± 0.62	4.40 ± 1.22^*∗∗*^
LDL/HDL	1.10 ± 0.32	1.36 ± 0.35	1.40 ± 0.41	1.67 ± 0.66

^*∗∗*^ *P* < 0.01.
