# Supplementary material for: The Association of Adiposity Indices and Plasma Vitamin D in Young Females in Saudi Arabia
Source: Int J Endocrinol. 2016 Jul 25;2016:1215362. doi: 10.1155/2016/1215362 (PMC4976256; doi:10.1155/2016/1215362)
Supplement: Supplementary file 1 — This is Vitamin D questionnaire submitted to all subjects to estimate the intake of vitamin D rich food articles, sun exposure, and physical activity. The questionnaire is designed to provide cummulative scores for each segment, however the scores were not visible to the subject. [file 1215362.f1.docx]

**VITAMIN D AND CALCIUM QUESTIONNAIRE**

1. **Demographic**:

Name __________________________ Today’s Date ______________

Age ________________ Phone ______________________________

College __________________________ level_____________________

Marital status ___________________________ Parity______________

**B. Diet**

1. Do you drink glasses of milk (hot or cold)? If yes, how often, or How much milk would you typically drink?

| Frequency | score |
| --- | --- |
| o No | 1 |
| o 2-3 times / month | 2 |
| o 10 times / month (1-3/weeks) | 3 |
| o 20 times / month (4-6/weeks) | 4 |
| o 30 times / month (once a day) | 5 |
| o > 30 times/month ( ≥ 2 times / day or more) | 6 |

2. Do you usually have milk in tea or coffee?

| Frequency | score |
| --- | --- |
| o No | 1 |
| o 2-3 times / month | 2 |
| o 10 times / month (1-3/weeks) | 3 |
| o 20 times / month (4-6/weeks) | 4 |
| o 30 times / month (once a day) | 5 |
| o > 30 times/month ( ≥ 2 times / day or more) | 6 |

3. Do you eat breakfast cereal with milk?

| Frequency | score |
| --- | --- |
| o No | 1 |
| o 2-3 times / month | 2 |
| o 10 times / month (1-3/weeks) | 3 |
| o 20 times / month (4-6/weeks) | 4 |
| o 30 times / month (once a day) | 5 |
| o > 30 times/month ( ≥ 2 times / day or more) | 6 |

Total milk score: 18

4. Do you usually eat yoghurt? >>>>>>>

| Frequency | score |
| --- | --- |
| o No | 1 |
| o 2-3 times / month | 2 |
| o 10 times / month (1-3/weeks) | 3 |
| o 20 times / month (4-6/weeks) | 4 |
| o 30 times / month (once a day) | 5 |
| o > 30 times/month ( ≥ 2 times / day or more) | 6 |

5. Do you usually eat butter/oil / olive oil?

| Frequency | score |
| --- | --- |
| o No | 1 |
| o 2-3 times / month | 2 |
| o 10 times / month (1-3/weeks) | 3 |
| o 20 times / month (4-6/weeks) | 4 |
| o 30 times / month (once a day) | 5 |
| o > 30 times/month ( ≥ 2 times / day or more) | 6 |

6. Do you usually eat cheese (all kinds)?

| Frequency | score |
| --- | --- |
| o No | 1 |
| o 2-3 times / month | 2 |
| o 10 times / month (1-3/weeks) | 3 |
| o 20 times / month (4-6/weeks) | 4 |
| o 30 times / month (once a day) | 5 |
| o > 30 times/month ( ≥ 2 times / day or more) | 6 |

7. Do you usually eat bread, wraps, and/or rolls?

| Frequency | score |
| --- | --- |
| o No | 1 |
| o 2-3 times / month | 2 |
| o 10 times / month (1-3/weeks) | 3 |
| o 20 times / month (4-6/weeks) | 4 |
| o 30 times / month (once a day) | 5 |
| o > 30 times/month ( ≥ 2 times / day or more) | 6 |

8. Do you usually eat meat?

| Frequency | score |
| --- | --- |
| o No | 1 |
| o 2-3 times / month | 2 |
| o 10 times / month (1-3/weeks) | 3 |
| o 20 times / month (4-6/weeks) | 4 |
| o 30 times / month (once a day) | 5 |
| o > 30 times/month ( ≥ 2 times / day or more) | 6 |

9. Do you usually eat eggs (raw, cooked, in dishes eg: omelette)?

| Frequency | score |
| --- | --- |
| o No | 1 |
| o 2-3 times / month | 2 |
| o 10 times / month (1-3/weeks) | 3 |
| o 20 times / month (4-6/weeks) | 4 |
| o 30 times / month (once a day) | 5 |
| o > 30 times/month ( ≥ 2 times / day or more) | 6 |

10. Do you usually drink carbonated drinks?

| Frequency | score |
| --- | --- |
| o No | 6 |
| o 2-3 times / month | 5 |
| o 10 times / month (1-3/weeks) | 4 |
| o 20 times / month (4-6/weeks) | 3 |
| o 30 times / month (once a day) | 2 |
| o > 30 times/month ( ≥ 2 times / day or more) | 1 |

11. Do you usually consume beverages fortified with vitamin D and/or calcium?

| Frequency | score |
| --- | --- |
| o No | 1 |
| o 2-3 times / month | 2 |
| o 10 times / month (1-3/weeks) | 3 |
| o 20 times / month (4-6/weeks) | 4 |
| o 30 times / month (once a day) | 5 |
| o > 30 times/month ( ≥ 2 times / day or more) | 6 |

Total Maximum Diet score: 61

1. **Sunlight Exposure**

12. On Average, during the past 12 months, approximately how many minutes a day have >>>you spent outdoors in the sun between 10:00 AM and 2:00 PM .

a) Head and hands > > o None (0) o 5-15min/day (1) o 15-30 min/day (2) o more than 30 min/day (3)

b) arms >> o None (0) o 5-15min/day (1) o 15-30 min/day (2) o more than 30 min/day (3)

c) legs >> o None (0) o 5-15min/day (1) o 15-30 min/day (2) o more than 30 min/day (3)

13. During the past 12 months , have you received any UV exposure from indoor tanning >>>>equipment ? >>>>o Yes (1) o No (0)

Total Maximum Score of sun exposure: 9

1. **Physical Activity**

14. On average over the past year , how often did you watch TV or play video games “ screen >>>Time “ ?

| Hours of TV or video | None | Less than 1 hour a day | 1 to 2 hours a day | 2 to 3 hours a day | More than 4 hours a day |
| --- | --- | --- | --- | --- | --- |
| On weekday before 6 pm | 4 | 3 | 2 | 1 | 0 |
| On weekday after 6 pm | 4 | 3 | 2 | 1 | 0 |
| On weekend before 6 pm | 4 | 3 | 2 | 1 | 0 |
| On weekend after 6 pm | 4 | 3 | 2 | 1 | 0 |

Total maximum score: 16

15. Over the past 12 months , how many times do climb up stairs each day at home (approx. 10 >>>>>>step)?

| Number of times you climbed up stairs at home | Average over the last 12 months | | | | | |
| --- | --- | --- | --- | --- | --- | --- |
|  | None | 1 to 5 times a day | 6 to 10 times a day | 11 to 15 times a day | 16 to 20 times a day | More than 20 times a day |
| On a weekday | 0 | 1 | 2 | 3 | 4 | 5 |
| On a weekend day | 0 | 1 | 2 | 3 | 4 | 5 |

Total maximum score: 10

16. Approximately how many hours do spend doing activities in and around your home?

| Approximate number of hours each week | Average over the past 12 months | | | | | | |
| --- | --- | --- | --- | --- | --- | --- | --- |
|  | None | Less than 1 hours a week | 1 to 3 hours a week | 3 to 6 hours a week | 6 to 10 hours a week | 10 to 15 hours a week | More than 15 hours a week |
| Preparing food, cooking and washing up | 0 | 1 | 2 | 3 | 4 | 5 | 6 |
| Shopping for food and | 0 | 1 | 2 | 3 | 4 | 5 | 6 |
| Shopping and browsing in shops for other items (e.g. clothes, toys) | 0 | 1 | 2 | 3 | 4 | 5 | 6 |
| Cleaning the house | 0 | 1 | 2 | 3 | 4 | 5 | 6 |
| Doing the laundry and ironing | 0 | 1 | 2 | 3 | 4 | 5 | 6 |
| Caring for pre-school children or babies at home (not as paid employment) | 0 | 1 | 2 | 3 | 4 | 5 | 6 |
| Caring for handicapped elderly or disabled people at home (not as paid employment) | 0 | 1 | 2 | 3 | 4 | 5 | 6 |

Total maximum score; 42

17. During the last week, how many hours did you spend on each of the following activities?

|  | None | Some but less than 1 hour | 1 hour but less than 3 hours | 3 hours or more |
| --- | --- | --- | --- | --- |
| Physical exercise such as swimming, jogging, aerobics, football, tennis, gym workout etc. | 0 | 1 | 2 | 3 |
| Walking including walking to work, shopping, for pleasure, etc. | 0 | 1 | 2 | 3 |
| Others | 0 | 1 | 2 | 3 |

Total score; 9

18. How would you describe your usual walking pace? Please mark one box only

| Slow pace (less than 3 mph) | Steady average pace | Fast pace (over 4 mph) | Brisk pace |
| --- | --- | --- | --- |
| 1 | 2 | 3 | 4 |

Total maximum physical activity score: 81

1. **Past Medical & Family History**

19. Do you suffer from any disease?

o Yes o No

If yes, please list them

________________________________________________

20. Do you take any medications?

o Yes o No

If yes, please list them

_________________________________________________________

21. Family history :

o diabetes o hypertension o hyperlipidemia o obesity o cardiovascular o others _________________________________________________________
